# Controllable quantum scars induced by spin–orbit couplings in quantum dots

**DOI:** 10.1186/s11671-024-04015-7

**Published:** 2024-04-29

**Authors:** Lin Zhang, Yutao Hu, Zhao Yao, Xiaochi Liu, Wenchen Luo, Kehui Sun, Tapash Chakraborty

**Affiliations:** 1 School of Physics, Central South University, Changsha, 410083 China; 2 Department of Physics and Astronomy, University of Manitoba, Winnipeg, R3T 2N2 Canada

## Abstract

Spin–orbit couplings (SOCs), originating from the relativistic corrections in the Dirac equation, offer nonlinearity in the classical limit and are capable of driving chaotic dynamics. In a nanoscale quantum dot confined by a two-dimensional parabolic potential with SOCs, various quantum scar states emerge quasi-periodically in the eigenstates of the system, when the ratio of confinement energies in the two directions is nearly commensurable. The scars, displaying both quantum interference and classical trajectory features on the electron density, due to relativistic effects, serve as a bridge between the classical and quantum behaviors of the system. When the strengths of Rashba and Dresselhaus SOCs are identical, the chaos in the classical limit is eliminated as the classical Hamilton’s equations become linear, leading to the disappearance of all quantum scar states. Importantly, the quantum scars induced by SOCs are robust against small perturbations of system parameters. With precise control achievable through external gating, the quantum scar induced by Rashba SOC is fully controllable and detectable.

**Supplementary Information:**

The online version contains supplementary material available at 10.1186/s11671-024-04015-7.

## Introduction

Quantum scars which manifest as the localization behavior displaying certain unstable classical periodic orbits exist in the high-energy levels in the quantum system with chaotic dynamics being driven in its classical limit. The quantum scar was first discovered while studying the quantum eigenstates of the stadium billiard model which drives chaotic dynamics in the corresponding classical model [1, 2] and later was named as such by Heller [3]. Quantum scarring has thus far drawn great attention and interest [4–7] and has been observed experimentally in various systems, including quantum well and microwave resonators [8–13]. The localization nature of quantum scarring without participation of the many-body system is convenient to be applied and attracts interest across various fields. On the other hand, the quantum many-body scars localizing eigenstates to prevent thermalization are expected to be useful in quantum computing [14–19].

Recently, the perturbation induced quantum scars have been studied in quantum dot (QD) systems confined at the semiconductor heterostructure with or without an external magnetic field [20–22]. These quantum scars are induced by a bunch of impurities which make the (nearly) degenerate states of the QD resonant to localize the electron density along the underlying classical trajectories. As an artificial atom [23], the low-dimensional QD [24–28] offers an ideal platform for controlling both the spin and the charge of single or multiple electrons. The parabolic confinements of QDs render the system a two-dimensional (2D) quantum harmonic oscillator which holds practical and fundamental significance in physics. The quantum scars found in QDs also reveal profound connections between the classical and the quantum systems.

Both nonrelativistic and relativistic quantum systems have been found to possess quantum scars [29, 30]. The focus has also been on quantum scarring in relativistic quantum systems which are described by the Dirac equation, especially in graphene systems [31–34]. However, the experiments in monolayer and bilayer graphene to explore quantum chaos have not been successful [35]. On the other hand, the spin–orbit coupling (SOC) is also a relativistic effect originating from the Dirac equation. Its corresponding classical Hamiltonian leads to nonlinearity in Hamilton’s equation and it is possible to drive chaotic dynamics [36–38]. Exploring quantum scars induced by SOCs could thus offer an intriguing avenue [39].

The studies on QDs with Rashba SOC or/and Dresselhaus SOC have been reported extensively thus far [40–56]. The ground states of QDs with SOCs have been studied to explore topological nontrivial features in spin fields [57–60]. Vortex-like spin textures in the ground states carry different topological charges induced by Rashba SOC or linear Dresselhaus SOC. Considering that the Rashba SOC can be conveniently tuned via an external gate [61–65], the spin textured ground states could have potential applications in spintronics and quantum information [66–68]. Yet, the excited states in QDs with SOCs have not been sufficiently studied, especially in the energy region containing classical chaos.

Here we investigate the excited states as well as the quantum scarring in spin–orbit coupled QDs. The scars can appear in the eigenstates quasi-periodically (the period is not fixed and gradually increased with the eigenenergy). We also confirm that the condition of scarring in the quantum states exactly follows the chaos condition in the classical limit. When the strengths of the Rashba and the Dresselhaus SOCs are equal, the classical Hamilton’s equations are linear and no longer lead to chaos, hence there is no scar in the quantum system. Otherwise we observe various quantum scars depending on the systematic parameters. It is worth mentioning that the quantum scars induced by SOCs in QDs are highly robust against with small perturbations, unlike the classical chaotic behavior and could be referred to its quantum feature that the energies are discrete. Comparing with the impurities induced quantum scars, the scars induced by SOCs are more tunable, less random, exist at low-energy levels, and spin-involved. We thus expect the corresponding measurements to be more convenient by scanning tunneling spectroscopy [69, 70], scanning gate microscopy [71], scanning the NMR experiment [72–74] and the spin-dependent transport [39, 75–77].

## Model and formula

The Hamiltonian of the quantum dot with both the Rashba and Dresselhaus SOCs is given by

1 $$\begin{aligned}{} & {} {\mathcal {H}}=\frac{{\textbf{P}}^{2}}{2m^{*}}+\frac{m^{*}}{2}\left( \omega _x^2x^2+\omega _y^2y^2\right) +\frac{\Delta }{2}\sigma _z^{}+{\mathcal {H}}^{}_{SOC}, \end{aligned}$$

2 $$\begin{aligned}{} & {} {\mathcal {H}}^{}_{SOC}=g_1^{}\left( \sigma _x^{}P_y^{}-\sigma _y^{}P_x^{}\right) +g_2^{}\left( \sigma _y^{}P_y^{}-\sigma _x^{}P_x^{}\right) , \end{aligned}$$

where $\omega ^{}_x$ and $\omega ^{}_y$ describe the parabolic confinements in the *x* and *y* dimensions, respectively. $\sigma ^{}_i$ is the Pauli matrix and the strengths of the Rashba and Dresselhas SOCs are $g_1^{}$ and $g_2^{}$ respectively. $P_i^{}=p_i^{}+eA_i^{}$ is the kinetic momentum, where *e* is the charge of an electron and the vector potential can be chosen in the symmetric gauge ${\textbf{A}}=\frac{1}{2} B\left( -y,x,0\right)$ with the magnetic field *B*. The Zeeman term, which is the first order correction of the relativistic effect, is $\Delta =g\mu _B^{}B$, where *g* is the Landé factor and $\mu _B$ is the Bohr magneton.

In an adiabatic model the SOCs could have a classical correspondence [36–38] in the absence of a magnetic field,

3 $$\begin{aligned} {\mathcal {H}}_{SOC}= & {} (g_1 p_y - g_2 p_x) \sigma _x + (g_2 p_y - g_1 p_x) \sigma _y \nonumber \\ \rightarrow H^C_{SOC}= & {} - \sqrt{(g_1 p_y - g_2 p_x) ^2 + (g_2 p_y - g_1 p_x) ^2}, \end{aligned}$$

which provides nonlinearity and is able to drive chaotic dynamics in the system. The full classical Hamiltonian reads

4 $$\begin{aligned} H^C= & {} \frac{p_x^2 + p_y^2}{2m^*}+ \frac{m^*}{2} (\omega _x^2 x^2 + \omega _y^2 y^2) \nonumber \\{} & {} \quad - \sqrt{g_1^2+g_2^2} \sqrt{p_x^2+p_y^2 -\frac{4g_1 g_2 }{g_1^2+g_2^2}p_x p_y }. \end{aligned}$$

By solving the canonical equations, chaotic dynamics can appear when the SOC is anisotropic. If there is only Rashba SOC present, then $H^C_{SOC}=-g_1\sqrt{ p_x^2 + p_y^2}$. On the other hand, if only Dresselhaus SOC is present, then the classical correspondence is the same as that of Rashba SOC. It implies that whichever SOC is present, the classical behavior remains the same. Note that if the confinement trap is isotropic, classical trajectories in the phase space would be regular. The way leading to chaotic dynamics is to make the confinement anisotropic, which effectively makes the SOC anisotropic in the classical limit. Once chaos appears, the corresponding quantum scar induced by the SOC should be observed in the quantum dot. Considering the classical correspondence of the two types of SOCs being the same, the quantum scar would also be identical.

The system is highly tunable, as both the Rashba SOC and confinements can be tuned by external gates, and the ratio of the Rashba SOC to the Dresselhaus SOC can be modified by applying an in-plane magnetic field [65]. It is worth mentioning a special case where $g_1= \pm g_2$, i.e. the two SOCs are present simultaneously with equal strength. The classical correspondence becomes $H^C_{SOC}=-g_1 (p_x - p_y)$, which is a linear term in the Hamiltonian and does not lead to chaos.

To study the quantum scar of the quantum dot system described by the Hamiltonian in Eq. (1), the eigenstates are calculated in the exact diagonalization scheme. The Hamiltonian matrix is constructed in the basis of the two dimensional (2D) quantum oscillator whose Hamiltonian is $H_{0}=\frac{{\textbf{p}}^{2}}{ 2\,m^{*}}+\frac{m^{*}}{2} \left( \Omega _{x}^{2}x^{2}+\Omega _{y}^{2}y^{2}\right) +\frac{\Delta }{2}\sigma _{z}$, where the renormalized frequency is defined as $\Omega _{x,y}= \sqrt{\omega _{x,y}^2+ \omega _c^2/4}$ with the cyclotron frequency in a magnetic field being given by $\omega _c = |e|B/m^*$. The basis of the 2D quantum oscillator is $|n \rangle \equiv |n_x,n_y, n_s \rangle$ where *n* is a collective index marking the number of the basis, $n_s$ is the spin index in *n*, and $n_x$ and $n_y$ denote the two quantum numbers in two directions of the 2D quantum oscillator, respectively. The associated wave function of this basis is

5 $$\begin{aligned} \psi _{n_{x},n_{y}}\left( {\textbf{r}}\right) =\frac{\exp \left( -\frac{ x^{2}}{2\ell _{x}^{2}}-\frac{y^{2}}{2\ell _{y}^{2}}\right) }{\sqrt{ 2^{n_{x}+n_{y}}n_{x}!n_{y}!\pi \ell _{x}\ell _{y}}}H_{n_{x}}\left( \frac{x}{ \ell _{x}}\right) H_{n_{y}}\left( \frac{y}{\ell _{y}}\right) , \end{aligned}$$

where the natural lengths in the two directions are $\ell _{x,y} =\sqrt{\hbar /m^*\Omega _{x,y}}.$ In principle, there is no upper limit of $n_{x,y}$, so that the matrix of the Hamiltonian is infinity large. Practically, a truncation of $n_{x,y}$ is necessary and the low-energy states can be found accurately.

Once the Hamiltonian (1) is diagonalized, the $m-$th eigenstate can be expressed by the basis, $|\Psi _m \rangle = \sum _n C_n^m |n \rangle$, and its wave function is $\Psi _m ({\textbf{r}}) = \langle {\textbf{r}}| \Psi _m \rangle = \sum _n C^m_n \psi _{n_x, n_y}({\textbf{r}}) | n_s\rangle$. Here, $|n_s \rangle$ represents an eigenstate of $\sigma _z$, and thus the wave function $\Psi _m ({\textbf{r}})$ is a two-component spinor. Generally, any observable field is given by

6 $$\begin{aligned} \Lambda ({\textbf{r}}) =\Psi _m^\dag ({\textbf{r}}) \Lambda \Psi _m ({\textbf{r}}), \end{aligned}$$

where $\Lambda$ is the corresponding operator including the density operator *n* (unity matrix), spin operators $\sigma _\mu$ with $\mu =x,y,z$ and etc. The current fields which are related to the spin fields are defined by $j_{x}\left( {\textbf{r}}\right) =-\frac{e}{m^{*}} \text {Re} \left[ \Psi _m ^{\dag }\left( {\textbf{r}}\right) P_{x}\Psi _m \left( {\textbf{r}}\right) \right] +eg_{1}\sigma _{y}\left( {\textbf{r}}\right) +eg_{2}\sigma _{x}\left( {\textbf{r}}\right)$ and $j_{y}\left( {\textbf{r}}\right) =-\frac{e}{m^{*}}\text {Re}\left[ \Psi ^{\dag }\left( {\textbf{r}}\right) P_{y}\Psi \left( {\textbf{r}}\right) \right] -eg_{1}\sigma _{x}\left( {\textbf{r}}\right) -eg_{2}\sigma _{y}\left( {\textbf{r}}\right)$.

Without loss of generality, we consider here the InAs quantum dots with the material parameters: the effective mass of electron is $m^{*}=0.042m_e$ where $m_e$ is the mass of free electron and Landé factor $g=-14$. The size of the QD is not fixed here, but we could fix the confinement in *x* direction and vary the other confinement. The confinement lengths are defined by $R_i= \sqrt{\hbar / m^* \omega _i}$ and $R_x$ is fixed to 30 nm associated with the characterized confinement energy $\hbar \omega _x = 2$ meV.

## Results

### Isotropic QD with a single SOC

When only one SOC is present and the quantum dot is isotropic, $\omega _x=\omega _y=\omega$, the associated classical Hamiltonian is $H^C= \frac{p_x^2 + p_y^2}{2\,m^*}+ \frac{m^* \omega ^2}{2} ( x^2 + y^2) - g_{1(2)} \sqrt{p_x^2 + p_y^2},$ which does not lead to chaos [36–38]. In the quantum regime, the densities and the spin fields of all eigenstates in the isotropic quantum dot are deformed by the SOC. The rotational symmetry does not only exist in the ground state, but also exists in all excited states of the single-particle system, due to the symmetry $[L_z \pm \sigma _z/2, {\mathcal {H}}]=0$, where $L_z$ is the *z* component of the angular momentum [57]. The topological feature of the spin fields is also retained in the excited states, i.e. nontrivial patterns with nonzero topological charges are textured by the SOC.

**Fig. 1:**
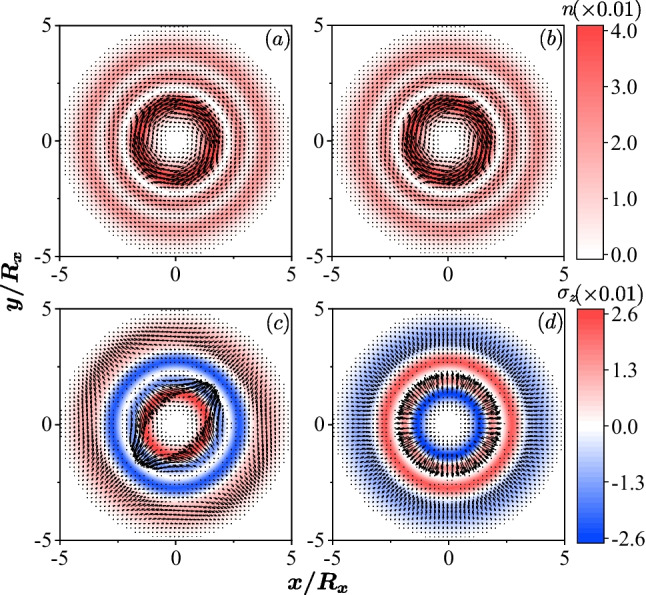
The density profiles and the spin fields of the 100th eigen states in an isotropic QD ($R_x=R_y=30$ nm) with different SOCs, in the absence of external magnetic field. Panels **a** and **c** are for the QD with Dresselhaus SOC $\hbar g_2=40$ nm$\cdot$meV, while panels **b** and **d** are for the QD with Rashba SOC $\hbar g_1=40$ nm$\cdot$meV. In **a** and **b**, the color represents the density of the electron and the arrows represent the current vector $(j_x({\textbf{r}}), j_y({\textbf{r}}))$. **c** and **d**: The color stands for $\sigma _z ({\textbf{r}})$ and the arrows for the in-plane spin fields $(\sigma _x({\textbf{r}}), \sigma _y({\textbf{r}}))$ with topological charge $-1$ and 1, respectively. All the observable quantities are in units of $1/R_x^2$ hereafter

Our study indicates that the densities of all eigenstates have a circlular shape with topological nontrivial vortex-like spin textures (Fig. 1). The Rashba SOC induces a topological charge $+1$ of the in-plane spin field, while the Dresselhaus SOC leads to topological charge $-1$ [57, 58]. Further, the current fields of the two cases are also shown in Fig. 1, where the two SOCs lead to rotating currents with the same vorticity related to their spin fields.

When a perpendicular magnetic field is introduced, the electron has a cyclotron motion in the magnetic field. The densities of the eigenstates maintain a circular structure with rotational symmetry when only one SOC is present in an isotropic QD. However, the directions of the current may be changed by the magnetic field in different eigenstates.

### Isotropic QD with combination of different SOCs

The chaotic dynamics can be driven in the isotropic dot by combining the two SOCs arbitrarily and $|g_1| \ne |g_2|$. The classical Hamiltonian is

7 $$\begin{aligned} H^C= & {} \frac{\left( p_{x}^{\prime }\right) ^{2}+\left( p_{y}^{\prime }\right) ^{2}}{2m^*}+\frac{1}{2}m^* \omega ^{2}\left( x^{2}+y^{2}\right) \nonumber \\{} & {} \quad -\sqrt{\left( g_{1}+g_{2}\right) ^{2}\left( p_{x}^{\prime }\right) ^{2}+\left( g_{1}-g_{2}\right) ^{2}\left( p_{y}^{\prime }\right) ^{2}}, \end{aligned}$$

where $p'_x = (p_y - p_x)/\sqrt{2}$ and $p'_y = (p_x + p_y)/\sqrt{2}$. It is obvious that the Hamiltonian governs a linear system only when $g_1 = \pm g_2$, since its canonical equations are linear. Otherwise, the canonical equations are nonlinear and such systems are possible to hold the chaotic dynamics. The isotropically confined QD becomes to an anisotropic system due to the arbitrary mixing of the two SOCs. This implies that, in the quantum regime, the quantum scars which is represented by the electron density localizing along the classical trajectory can appear in the excited states. The absence of the magnetic field conserves the time reversal symmetry and the quantum scar states appear in pair due to the Kramers pair.

**Fig. 2:**
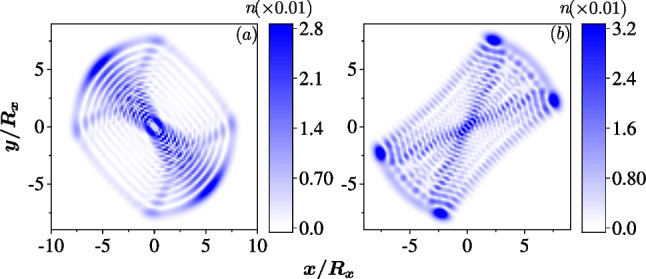
The quantum scar states in an isotropic QD ($R_x=R_y=30$ nm) with mixing of the two SOCs, $\hbar g_1=40$ nm$\cdot$meV and $\hbar g_2 =10$ nm$\cdot$meV. In **a** and **b**, the color represents the densities of the electron $n({\textbf{r}})$ in the 1028th and 1247th eigenstates, respectively

In Fig. 2, we show two quantum scars in the excited states of an isotropic QD with a combination of Rashba and Dresselhaus SOCs, $\hbar g_1=40$ nm$\cdot$meV and $\hbar g_2=10$ nm$\cdot$meV. In Fig. 2a, the density of electron is localized to an axe-shape pattern, while an ‘X’-trajectory appears in Fig. 2b. These patterns are different from the array-shaped density profiles of states in a QD without SOC significantly (i.e. the densities observed in a 2D quantum oscillator).

### Quantum Lissajous scar in anisotropic dot induced by a single SOC

Another quantum scar, which is called quantum Lissajous scar [22], can emerge in an anisotropic QD where the ratio of the 2D confinements $\omega _x/\omega _y$ is a rational number. The two confinement potentials are accessible to be manipulated via gates. The original idea to realize the quantum Lissajous scars is by the massive random impurities which induce chaos and mix different eigenstates of the basis. The scar indicates the classical behavior of an anisotropic 2D oscillator, so that the density of the electron of the quantum scar state localizes around the Lissajous curve corresponding to the ratio $\omega _x/ \omega _y$.

**Fig. 3:**
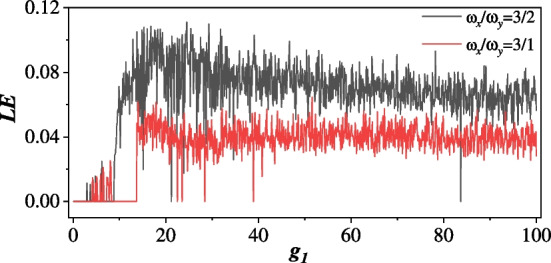
The largest LEs of the two anisotropic systems with $\omega _x/ \omega _y =3/1$ and 3/2. These LEs are calculated in the dimensionless Hamiltonian in Eq. (8) with varied $g_1$ and fixed $g_2=0$. The chaos of the system is related to $g_1$

In an anisotropic QD with Rashba or Dresselhaus SOC, the corresponding classical Hamiltonian also leads to chaotic dynamics in the phase space obtained by its Hamilton’s equation. For simplicity, the dimensionless Hamiltonian with $m^*=1$ is

8 $$\begin{aligned} H^C= p_x^2 + p_y^2 + \frac{1}{2} \omega _x^2 x^2 + \frac{1}{2} \omega _y^2 y^2 - g_{1(2)} \sqrt{p_x^2 + p_y^2}, \end{aligned}$$

without the vector potential, i.e. no magnetic field. The Lyapunov exponent (LE) is employed to estimate the oscillation modes under parameter variation. The largest LE being positive indicates the existence of a chaotic state, while the largest LE being negative denotes the system described by periodical states only. In Fig. 3, the largest LE [78, 79] of the two examples with $\omega _x / \omega _y =3/1,3/2$ demonstrate chaos in the system, when $g_2=0$ and $g_1$ is tuned (equivalent to tuning energy of the system). Note that for some $g_1$ the system shows no chaos.

**Fig. 4:**
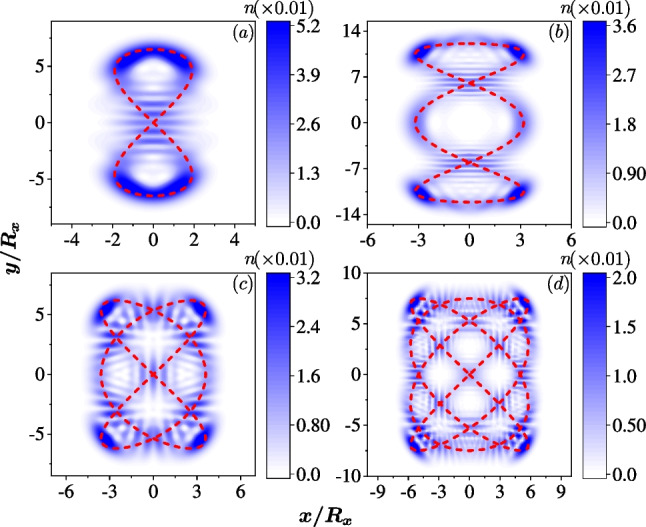
The quantum scar states in an anisotropic QD ($R_x=30$ nm) with only the Rashba SOC, $\hbar g_1=40$ nm$\cdot$meV. Colors represent for the density of the electron, $n({\textbf{r}})$. **a** The quantum scar in the 91st eigenstate around the Lissajous curve $\sim (\sin 2t, \sin t)$ since $R_y=\sqrt{2} R_x$ and $\omega _x / \omega _y =2/1$. **b** For the QD with $R_y=\sqrt{3} R_x$ and $\omega _x / \omega _y =3/1$, the quantum scar in the 535th eigenstate around the Lissajous curve $\sim (\sin 3t, \sin (t+\pi /2))$. **c** For the QD with $R_y=\sqrt{3/2} R_x$ and $\omega _x / \omega _y =3/2$, the quantum scar in the 331st eigenstate around the Lissajous curve $\sim (\sin 3t, \sin 2t))$. **d** For the QD with $R_y=\sqrt{4/3} R_x$ and $\omega _x / \omega _y =4/3$, the quantum scar in the 1404th eigenstate around the Lissajous curve $\sim (\sin 4t, \sin 3t))$. The dashed lines are the corresponding Lissajous curves drawn for guidance

We then demonstrate that the quantum Lissajous scars can be achieved by the relativistic correction, i.e., the SOC. In the quantum regime, the emerging quantum scars display the trajectory of a particle confined in a classical 2D oscillator. We first discuss the scars related to the specific closed Lissajous curves, $(x,y)\sim \left( \cos \eta _x t, \cos (\eta _y t+\frac{\pi }{2\eta _x}) \right)$, where $\omega _{x,y}=\eta _{x,y}\omega _0$. The open curve obtained by shifting the phase will be discussed in the next subsection. The quantum Lissajous scars for $\omega _x/ \omega _y=2/1, 3/1, 3/2, 4/3$ are shown in Fig. 4a–d, respectively. Around the cross points in the curves, the interference stripes are clearly visible, indicating both the classical and quantum features. In addition, the density profiles of the first 2000 states in different cases that the confinement ratios and the SOCs are varied are integrated into a video, which can be found in Additional file 1.

**Fig. 5:**
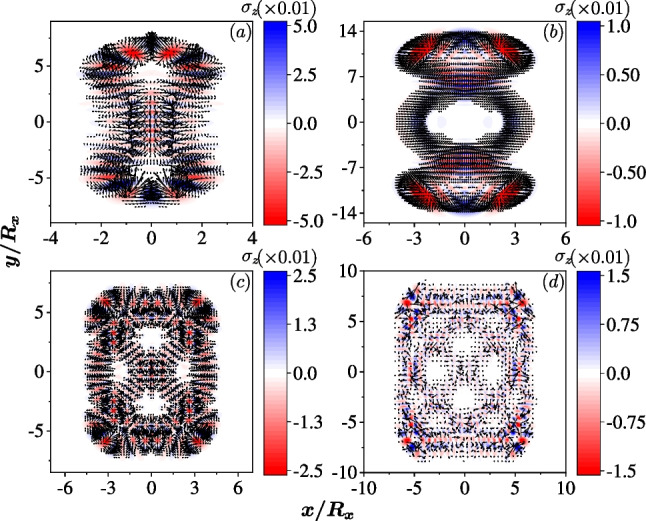
The spin fields of the quantum scar states in the anisotropic QD with the same systematic parameters as those used in Fig. 4. Colors represent $\sigma _z ({\textbf{r}})$ and the vectors represent the in-plane spin fields $(\sigma _x ({\textbf{r}}), \sigma _y ({\textbf{r}}))$

In Fig. 5a–d, we also indicate the associated spin fields of the four quantum Lissajous scars (Fig. 4a–d), respectively. Although the spin textures are somehow difficult to calculate analytically given that the perturbation calculations become complex and are not valid with a strong SOC, we can still numerically determine that the in-plane spins exhibit nontrivial patterns. There are numerous spin vortices localized and attached with the density profile, which are textured by the SOC.

It is worth noting that some eigenstates do not show any different density profile other than the regular dot-array patterns of the 2D quantum oscillator without the SOC. It is because in the corresponding energy region, the classical dynamics can be regular without chaos [36–38], resulting in the absence of quantum scar states. The chaotic behavior induced by the SOC differs significantly from that induced by random impurities, and so are the quantum scar states. Due to the randomness of the impurities sizes and locations, the quantum scar states therein can not be controlled or tracked precisely, and only the percentage of scar states among all eigenstates can be approximately estimated.

One cannot predict where the quantum scar states induced by impurities are, which makes detection of the scar states challenging. In contrast, in an anisotropic QD with SOC, the emerging quantum Lissajous scars are not random and can be accurately predicted. Each two quantum Lissajous scar states (due to the Kramers pair) appear quasi-periodically in a few eigenstates. For instance, in the case of $\omega _x/\omega _y=3/2$ with $\hbar g_1=40$ nm$\cdot$meV, the quantum scar states with density profile similar as those shown in Fig. 4c appear repeatedly in the (157th, 158nd), (167th, 168th), (177th, 178th), (189th, 190th), (199th, 200th) eigenstates, with a period of approximate 10 states between the two pairs of quantum Lissajous scar states. In higher energies, the Lissajous scar states appear in the (303rd, 304th), (317th, 318th), (331st, 332nd), (347th, 348th), (361st, 362nd), (377th, 378th) eigenstates. The separation between the two pairs of the quantum Lissajous scar states becomes about 14. The period is not fixed and will gradually increase (not monotonically) with increase of the energy.

Moreover, the quantum Lissajous scars induced by SOC can be found at very low energies, such as the ‘8’ shape Lissajous trajectory shown in Fig. 4a, which can be identified even down to the 15th eigenstate. More importantly, the Rashba SOC can be controlled by an external gate allowing for the manipulation of the quantum scar states. These characteristics of the quantum scars induced by SOC imply that SOC, especially the tunable Rashba SOC, greatly facilitates the measurement of the quantum scar state.

**Fig. 6:**
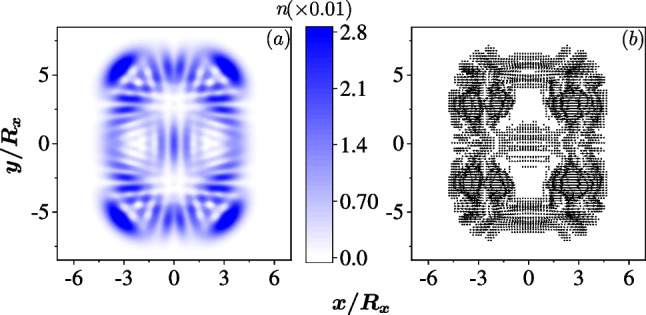
The quantum scar state in an anisotropic QD ($R_x=30$ nm and $R_y=\sqrt{3/2} R_x$) with the Rashba SOC, $\hbar g_1=40.4$ nm$\cdot$meV which is a little deviated from that used in Fig. 4c. **a** The quantum Lissajous scar in 331st eigenstate is the same (both the number of the eigenstate and the density profile) as that shown in Fig. 4c. **b** The current field of the 331st eigenstate

Considering the nature of the classical chaotic dynamics being sensitive to initial conditions, one might wonder if the quantum scar states are similarly sensitive to system parameters. If not, then the quantum scar states are more easily detected. We adjust the confinement ratio slightly, for instance, $\omega _x / \omega _y=3/2 \rightarrow 3.01/2$, and observe that the positions of the quantum scar states in all the eigenstates remain unchanged, as do their density profiles. We also examine the effect of adding a weak magnetic field, $B=0.05$ T. Although the Kramers’ degeneracy is lifted, the quantum scars persist in the same eigenstates as in the absence of the magnetic field, with only slight changes in density profiles. Similarly, when the SOC strength $g_1$ is slightly tuned, the positions or the density profiles of the quantum scar states remain unchanged. As illustrated in Fig. 6a, when the Rashba SOC is increased by one percent compare to that in Fig. 4c, namely $\hbar g_1=40.4$ nm$\cdot$meV, the quantum Lissajous scar does not change at all. We note that the current flow direction, displayed in Fig. 6b, does not align with the classical Lissajous trajectory, but is relevant to the spin fields shown in Fig. 5c. This character underscores the fundamental distinction between the classical behavior and the quantum mechanism.

The robustness of the quantum scar states against the external perturbations relies on the quantum properties of the system rather than its classical behavior. It can also be boiled down to the fact that small perturbations do not significantly alter the eigen energies of the eigenstates, allowing the corresponding classical behavior to remain within the chaos region, thus the scarring is frozen in the discrete-energy quantum system. This feature is also helpful for identifying the quantum scar states to make the possible measurement convenient.

Given that the classical Hamiltonians are identical for both Rashba and Dresselhaus SOCs, the density profiles of the quantum scar states induced by either one of the two SOCs are indistinguishable. Suppose that there are two QDs with the same confinement potentials, but one with Rashba SOC and the other with Dresselhaus SOC. The coupling strengths in the two QDs are identical, $g_1=g_2$. Our numerical studies indicate that the quantum scar states appear in the same position in the eigenstates of both cases, exhibiting exactly the same density profiles. However, the spin fields of these two states are different, providing a signature to distinguish the types of SOC.

### Lissajous curves pair scar

In anisotropic QDs with one SOC, the Lissajous patterns in open curves can also be found in scarring states, albeit with much lower probability. However, due to mirror symmetry, $x \rightarrow -x$ and $y \rightarrow -y$ without a magnetic field, a single open curve of the Lissajous pattern, which has lower symmetry, can not be found in any state. Instead, only a pair of Lissajous curves making up this symmetry emerges in a scarred state.

**Fig. 7:**
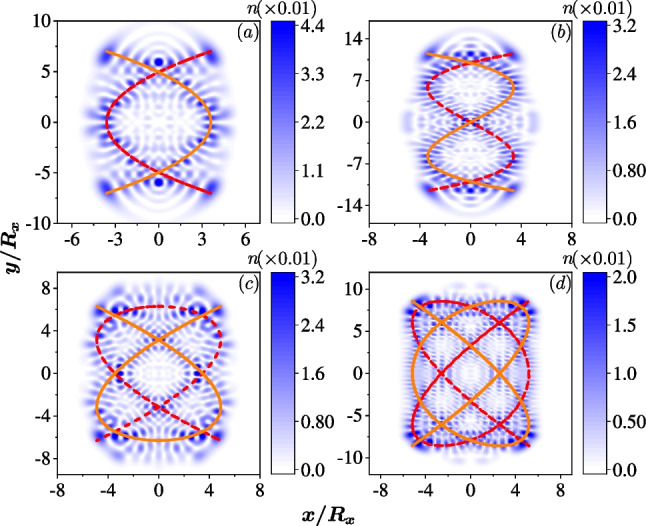
The Lissajous curves pairs in the quantum scar states in the anisotropic QD with the same systematic parameters as those used in Fig. 4. Colors represent the density of the electron. The quantum scars in **a** the 417th eigenstate with $\omega _x/\omega _y=2/1$, **b** the 659th eigenstate with $\omega _x/\omega _y=3/1$, **c** the 705th eigenstate with $\omega _x/\omega _y=3/2$, and **d** the 1571st eigenstate with $\omega _x/\omega _y=4/3$. The lines are the corresponding Lissajous curves drawn for guidance

In QDs with $\omega _x/\omega _y =2/1, 3/1, 3/2, 4/3$, the pairs of Lissajous curves in the quantum scars are illustrated in Fig. 7a–d, where the classical orbits $(x,y) \sim (\cos \eta _x t, \cos \eta _y t) + (\cos \eta _x t, \cos (\eta _y t+ \pi /\eta _x))$ are identified, respectively.

### Quantum regular states in anisotropic quantum dots

**Fig. 8:**
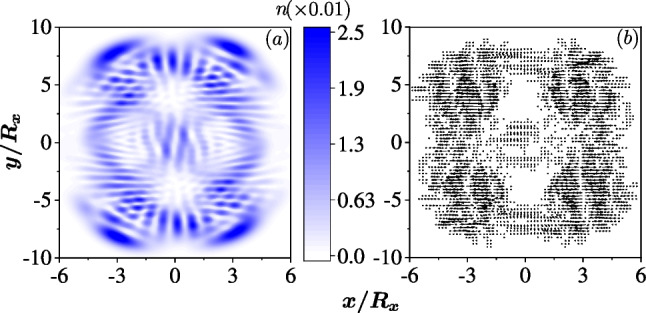
The quantum scar state in an anisotropic QD with $R_x=30$ nm and $R_y=\sqrt{3/2} R_x$. The two SOCs are all present, $\hbar g_1=40$ nm$\cdot$meV and $\hbar g_2=10$ nm$\cdot$meV. **a** The quantum Lissajous scar in 781st eigenstate is similar to that in Fig. 4**c**, but is a bit twisted. **b** The associated current field of this eigenstate

Finally, we discuss the effect of combining two SOCs in anisotropic QDs. As expected, when $g_1 \ne \pm g_2$, the quantum Lissajous scars appear. In Fig. 8, we show that the electron density forms the Lissajous curve in the 781st eigenstate, however, the Lissajous curve is not as regular as the case with only one SOC, and is slightly twisted, as does the corresponding current field.

**Fig. 9:**
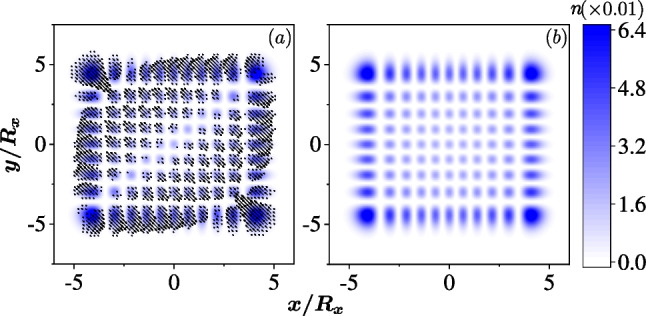
An example of array-like density in anisotropic QD with $R_x=30$ nm and $R_y=\sqrt{3/2} R_x$. The colors represent the density of the electron. The 390th eigenstate is selected for the cases **a** with equal Rashba and Dresselhaus SOCs $\hbar g_1=\hbar g_2=10$ nm$\cdot$meV, and **b** without SOC. The arrows in **a** represent the in-plane spin field of the state, while the in-plane spin field in **b** is zero

The special case that $g_1 = g_2$ has the classical correspondence,

9 $$\begin{aligned} H^C = \frac{\left( p_{x}^{\prime }\right) ^{2}+\left( p_{y}^{\prime }\right) ^{2}}{2m^*}+\frac{1}{2}m^* \omega _x^{2}x^{2}++\frac{1}{2}m^* \omega _y^{2} y^{2} -2g_1 p'_x, \end{aligned}$$

which describes a linear system without chaos. A similar Hamiltonian can be derived for $g_1=-g_2$. Thus for $g_1 = \pm g_2$ whether the QD is isotropic or anisotropic, no classical chaotic dynamics occur and no quantum scar appears. Our numerical calculation also confirms that all the density profiles of the eigenstates are alike dot-array, which are the same as the densities of the eigenstates of the QD without SOC, as shown in Fig. 9. The array-like densities are totally induced by the Hermite polynomials in the basis wave functions. The difference of the two cases is that the in-plane spin fields are nonzero in the QD with SOCs while the spin field is only polarized in the *z* direction in the QD without SOC.

When the external magnetic field is weak, the quantum Lissajous scars persist for $g_1 \ne \pm g_2$. However, when the magnetic field is increased, the scars are overwhelmed by the cyclotron motion. In the case of $g_1=\pm g_2$, due to the lack of chaotic dynamics, the densities of all the eigenstates form circles with rotational symmetry induced by the magnetic cyclotron motion in an isotropic dot. Nevertheless, the densities of the eigenstates remain arrays in an anisotropic QD when the magnetic field is weak, but evolve to elliptical shapes with increase of the magnetic field.

## Conclusion

In summary, we have studied the quantum scar states in quantum dots induced by relativistic effects, viz. the SOCs. For isotropic quantum dots, only the combination of Rashba and Dresselhaus SOCs can induce quantum scars, since the anisotropy and chaotic dynamics arise from the interplay between the two SOCs. In an anisotropic quantum dot, either one SOC or a combination of the two SOCs can lead to quantum Lissajous scar which may consist of one or a pair of Lissajous curves. We have to emphasize a special case where $g_1 = \pm g_2$ (the two SOCs have the same strength), which corresponds to a linear classical system without chaos. Thus, regardless of the confinement of the quantum dot, there is no quantum scar appearing in this case.

The quantum scars induced by SOCs are robust against small perturbations of the external conditions, such as small alterations in the confinement ratio $\omega _x / \omega _y$, weak magnetic fields, or variation in the strengths of the SOCs. The quantum Lissajous scars induced by SOCs emerge quasi-periodically in the eigenstates and can manifest at very low energies in particular. It implies that tuning the SOC is a stable and controllable way to obtain predictable quantum scars, unlike systems where quantum scars induced by a bunch of random impurities distribute randomly in the high-energy eigenstates. Given that the quantum scars discussed here appear in low-energy states and the direct observation of the orbit of the ground state of a quantum dot is already realized [80], our work paves the way to observe the quantum scars directly in such nanoscale systems, regardless of the materials, as long as the SOC is present. Furthermore, if direct observation is difficult currently, other indirect detection methods, such as spin polarization measurements, may also be useful due to the robustness of the associated quantum scars and the tunable property of the Rashba SOC. Especially, transport signals may be utilized to determine the scarring trajectory in quantum dot systems with SOCs, and spin-involved transport could prove beneficial for spintronics applications.

### Supplementary Information

Below is the link to the electronic supplementary material.

Supplementary file1 (MP4 127884 kb)

## Data Availability

All data supporting the conclusions of this article are included within the article.
